# Increased Micronuclei Frequency in Oral and Lingual Epithelium of Treated Diabetes Mellitus Patients

**DOI:** 10.1155/2018/4898153

**Published:** 2018-01-09

**Authors:** Jesús Emilo Quintero Ojeda, Maribel Aguilar-Medina, Vicente Olimón-Andalón, Rosa Alicia García Jau, Alfredo Ayala Ham, José Geovanni Romero Quintana, Erika de Lourdes Silva-Benítez, Guzmán Sanchez-Schmitz, Rosalío Ramos-Payán

**Affiliations:** ^1^Faculty of Odontology, Autonomous University of Sinaloa, 80010 Culiacán, SIN, Mexico; ^2^Faculty of Biological and Chemical Sciences, Autonomous University of Sinaloa, 80010 Culiacán, SIN, Mexico; ^3^Faculty of Biology, Autonomous University of Sinaloa, 80010 Culiacán, SIN, Mexico; ^4^Boston Children's Hospital and Harvard Medical School, Harvard University, Boston, MA 02115, USA

## Abstract

Diabetes mellitus (DM) is a metabolic disease characterized by persistent high levels of glucose in plasma. Chronic hyperglycemia is thought to increase oxidative stress and the formation of free radicals that in turn damage cells. Thus, we decided to determine the frequency of nuclear abnormalities in epithelial cells from cheek and tongue mucosa of DM patients with type 1 (DM1, treated only with insulin) and type 2 (DM2, treated with metformin) using the buccal micronucleus cytome (BMCyt) assay. Micronuclei frequency in cheek epithelial cells was higher in both DM1 (0.75 ± 0.31, *P* < 0.001) and DM2 (0.52 ± 0.27, *P* < 0.001) patients, as compared to healthy controls (0.07  ±  0.06). Similarly, micronuclei frequency in tongue epithelium was increased in DM1 (0.81  ±  0.22, *P* < 0.001) and DM2 (0.41  ±  0.21, *P* < 0.001) groups, in comparison to controls (0.06  ±  0.05). Besides, we found a positive correlation between micronuclei frequency and the onset time of DM2 in both cheek (*ρ* = 0.69, *P* < 0.001) and tongue epithelial cells (*ρ* = 0.71, *P* < 0.001), but not with onset time of DM1 or age of the patients. Considering all this, we pose that BMCyt could serve as a fast and easily accessible test to assess genotoxic damage during dental visits of DM patients, helping to monitor their disease.

## 1. Introduction

Diabetes mellitus (DM) is an endocrine metabolic disorder characterized by an abnormal elevated concentration of glucose in plasma (hyperglycemia) that when not treated can lead to ketoacidosis and chronic degenerative diseases of the heart, kidneys, eyes, and nerves. Besides self-resolving gestational DM, two other variants of DM can be defined based on insulin hormone: DM1 associated with low levels of insulin and DM2 associated with resistance to insulin. DM2, comprising almost 90% of DM cases, is the result of a combination of environmental and genetic factors [1–4]. DM prevalence has been increasing with more than 415 million cases worldwide and a mortality rate of 2.5–5 million each year [5, 6]. In Mexico, DM is the second cause of mortality with more than 400,000 cases diagnosed and more than 94,000 deaths, each year [7, 8]. Common dental problems associated with DM include xerostomia, increased cavities, periodontal disease, abscesses, dental loss, candidiasis, and taste loss [9–14]. Due the high prevalence of oral and dental problems on DM patients, dental health has taken a predominant place on DM care.

Chronic high levels of glucose during DM are thought to increase oxidative stress and the formation of free radicals that in turn damage cells [2, 15]. Reactive oxygen species (ROS) chemically attack cellular components altering metabolism, inflammatory mediators, and antioxidant defense mechanisms, overall favoring the pathogenesis of the disease and the persistence of genetic damage [16]. Micronucleus cytome assay is a technique used to determine smaller than normal nucleus and other nuclear abnormalities resulting from the incorrect splitting and sharing of genetic material replicated during mitosis due to external insults such as ionizing radiation or genotoxic substances [17]. Buccal micronucleus cytome (BMCyt) has become an important tool to monitor genetical damage commonly associated with chronic degenerative diseases and cancer [18–22]. This method relies on exfoliated cells rubbed off from the mouth instead of samples obtained in more invasive/less accessible manners such as blood draws, urine collection, skin stripping, and biopsies. BMCyt assay can determine the frequency of cells with micronuclei, pyknotic and lobulated nucleus, condensed chromatin, karyolysis, and karyorrhexis, where micronucleus is the main biomarker for chromosomal damage and instability [18]. Elevated micronuclei frequency has been found to be associated with DM2 [21], DM1 [23], elevated BMI [20], neuropathy [24], and nephropathy [25]. While elevated frequency of micronuclei in DM patients (including DM2) is commonly reported from blood cells [21], BMCyt assay has not been extensively applied to study damage to oral epithelium in DM patients [16, 26, 27]. Considering this, we decided to compare the frequency of micronuclei and other nuclear abnormalities observed in oral epithelium of cheek and tongue of DM1 (treated with insulin) and DM2 patients (treated with metformin, alone or in combination) and healthy individuals.

## 2. Materials and Methods 

### 2.1. Study Design and Population

Study design was comparative, transversal, and analytic. Sampling was not probabilistic, consisting of 10 DM1 patients, 40 DM2 patients, and 40 healthy subjects ethnically and geographically matched (Mexican Mestizos residents of the northwestern state of Sinaloa), including both genders, older than 18 years and having full medical and dental history. Anthropometric data included Body Mass Index (BMI, weight in kilograms divided by the square of height in meters), pathological familial antecedents, other systemic diseases, medication, and demographic and socioeconomic data. Subjects with hypercholesterolemia, arthritis, cancer, tobacco smoking, and alcoholism were excluded. DM diagnostic was performed by the endocrinology department of the Regional General Hospital (Culiacán Sinaloa, Mexico) and the dental care was performed at the Faculty of Odontology, Universidad Autónoma de Sinaloa (FO-UAS) (Culiacán, Sinaloa, Mexico). All DM patients were under glycemic control and monitored. Dental clinical history included tooth lost and its cause, previous dental treatments, dental prophylaxis, and use of oral hygiene aids (toothbrushes, dental floss, tongue cleaners, interdental cleaners, and mouth rinses). Mouth assessment included physical examination of lips, vestibular and palatal mucosa, salivary glands and tongue, and an odontogram. The study was approved by the Ethics Committee of FO-UAS and was performed in accordance with the Declaration of Helsinki. All subjects gave signed informed consent.

### 2.2. Sample Collection and Buccal Micronucleus Cytome (BMCyt) Assay

**Figure 1:**
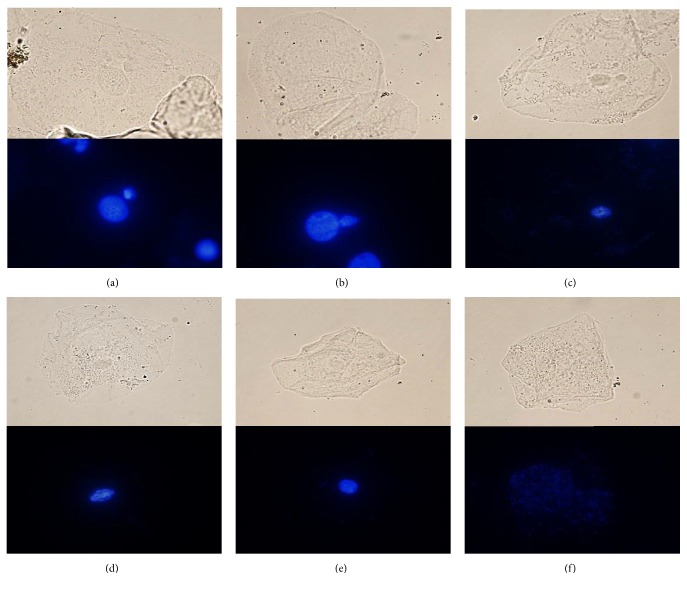
Representative confocal images of abnormal nuclei cells observed in the buccal micronucleus cytome assay. Nuclear abnormalities in BMCyt assay: micronucleus (a), lobulated nucleus (b), condensed chromatin (c), karyorrhexis (d), pyknotic nucleus, (e) and karyolitic cells (f).

Minimally invasive sampling was performed after rinsing mouth with water, as reported previously [28]. Exfoliated cells from cheek and tongue mucosa were carefully taken by robbing a cotton swap and extending cells over glass slides. Slides were dried at room temperature, fixed with methanol for 5 minutes and stored at 4°C until examination. The BMCyt assay was performed as reported elsewhere [18]. Briefly, fixed uncultured exfoliated oral mucosa cells mounted on glass slides were washed with distilled water and stained with Fluoroshield with DAPI (Sigma-Aldrich St. Louis, MO, USA). DAPI (4′,6-diamino-2-phenylindole) emits a strong fluorescence when bound to DNA. At least 2,000 cells per slide were analyzed for nuclear abnormalities using a Confocal Laser Scanning Microscope TCS SP8 (Leica, Wetzlar, Germany). The evaluated nuclear abnormalities are shown in Figure 1 and included micronucleus, lobulated nucleus, condensed chromatin, karyorrhexis, pyknotic nucleus, and karyolitic cells (Figures 1(a)–1(f)).

### 2.3. Statistical Analyses

All statistical analyses were performed with PASW software version 18.0 (SPSS inc., Chicago, IL, USA). Normal distribution was analyzed with Kolmogorov-Smirnov test. Variables from clinical history were cross-examined against DM1, DM2, and healthy subject groups. All results were expressed as the mean ± standard deviation or percentages, and differences between groups (controls versus DM1 or controls versus DM2) were tested by one-way variance analysis ANOVA with Bonferroni's posttest or with Chi-square (*χ*^2^). Variable's coefficient of correlation was determined by Pearson (*ρ*). Significance was defined at *P* < 0.05.

## 3. Results and Discussion

### 3.1. Clinical and Demographic Characteristics of Study Population

The characteristics of controls and patients are summarized in Table 1. Patients of this study were under glycemic control and monitored periodically, DM1 group received only insulin treatment, and DM2 were taking metformin alone (17.5%) or with glibenclamide (47.5%), plus captopril or enalapril (35.0%). The mean onset time of disease (years with diagnosis and treatment of diabetes) was similar for DM1 and DM2 patients (9.5 and 12.2 years, resp.). BMI analysis by gender found differences only in control and DM2 females (25.4 and. 33.9). According to the Centers for Disease Control and Prevention (CDC), a high BMI can be an indicator of high body fatness [29], and some studies had associated high BMI, blood pressure, and blood lipid status with hyperglycemia in female DM2 patients [30]. In agreement, our findings indicate that DM2 patients had a higher BMI than controls, especially women.

It has been reported that gender and age do not seem to influence cellular damage at oral epithelium [19]. In agreement, we do not find any correlation (*P* > 0.05 for Pearson's test) between nuclear abnormalities and gender or age of our patients. Since our sampling was not probabilistic we did not have a complete range of ages for all groups; however, the mean and range of ages between groups were similar and no significant differences were found (Table 1), arguing against any bias on the analysis.

There were no significant differences in active employment among the groups (Table 1). Regarding education level of control, DM1, and DM2 groups, respectively, they mainly completed elementary (30%, 30.0%, and 55%) and secondary school (35.0%, 40.0%, and 22.5%) in comparison to high school (25.0%, 30.0%, and 10%) and college (10.0%, 0.0%, and 2.5%); only DM2 group showed higher levels of analphabetism (10%). More than half of our patients only completed elementary school and this socioeconomic factor correlated with the low degree of knowledge, care, and management of the DM disease.

As expected, DMFT index was found to be of higher severity in DM patients as compared to controls. Mouth rinsing, dental flossing, and brushing cleaning after eating were practiced more frequently by the healthy group than DM patients. Consumption of sugar-sweetened beverages was also more frequent in controls (Table 1). While no significant differences were found between groups at odontogram analysis, DM patients showed dental alterations of higher severity than those observed in controls.

### 3.2. Cellular Damage in Cheek and Tongue Mucosa

Hyperglycemia in DM2 has been associated with an increased risk of DNA damage [31] due to downregulation of the DNA repair system [32] and the accumulation of oxidative stress parameters [33–35]. However, Grindel et al. had reported no significant differences in oxidative stress parameters, antioxidant enzyme activities, damage to DNA, and base excision repair capacity, neither between hyperglycemic DM2 patients, defined by hemoglobin A1c (HbA1c) cut-off/>7.5%, nor between diabetes duration (onset time), arguing that it might be due to good medical treatment with regular health checks in DM2 patients in Austria [30]. While DNA damage itself does not necessarily result in a phenotypic outcome, nuclear abnormalities are more likely to do so. In fact, BMCyt assay that determines the frequency of cells with abnormalities, chromosomal damage, and instability is used to monitor phenotypical progression of chronic degenerative diseases and cancer [18–22, 36, 37].

**Figure 2:**
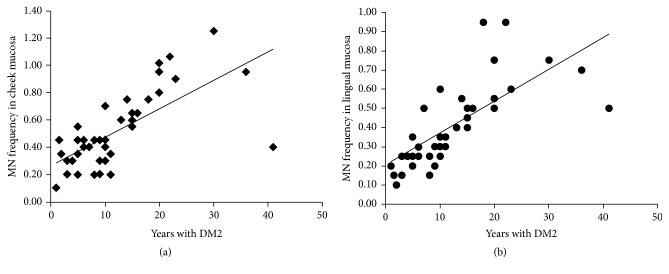
Correlation between percentage of micronuclei in cheek (a) and lingual (b) mucosa of DM2 and time with the disease.

Overall, our results showed higher frequencies of nuclear abnormalities (micronucleus, lobulated nucleus, condensed chromatin, karyorrhexis, pyknotic nucleus, and karyolitic cells) in cheek and tongue mucosa of almost all DM1 and DM2 patients as compared to controls (Table 2). The comparative analysis of these results by gender showed the same tendencies for all nuclear abnormalities in male and female groups, for both cheek and tongue. When nuclear abnormalities frequencies were analyzed against the onset time of the disease (years with diagnosis and treatment of diabetes), micronuclei in DM2 patients showed a positive correlation with diabetes duration (Figure 2), for both cheek (*ρ* = 0.69, *P* < 0.001) and tongue (*ρ* = 0.71, *P* < 0.001), but no correlation was observed for disease duration in DM1 group.

Interestingly, Pereira et al. studied micronucleus in prediabetes subjects showing that glycated hemoglobin levels do not correlate with micronuclei frequency [38]. Since there was no treatment, this work supports the notion that disease duration and hyperglycemia are required for genetic damage. In contrast, Grindel et al. argued that most studies compare DNA damage of DM2 patients to healthy controls but disregard the fact that, within its progression, DM2 is a considerably diverse disease requesting different medical treatment approaches leading to a broad range of hyperglycemia levels [30]. Some reports have argued that metformin, commonly prescribed for DM2 [39], could have genotoxic potential either alone or in combination with other therapeutics [2, 40]. In contrast, there are studies showing that short-term exposure to metformin does not increase micronuclei in human cells [41, 42]. There are oral drugs such as glibenclamide that report no increase of micronuclei frequencies in treated DM2 patients [43]. Similarly, there is not a study demonstrating any direct role of insulin treatment on micronuclei induction on DM1 patients [23, 44–47].

In this regard, our study was designed to correlate diabetes (the disease condition itself and its onset time) with frequencies of nuclear abnormalities, by including both DM1 (treated with insulin) and DM2 (treated with metformin alone or in combination) patients. The inclusion of DM1 group was useful to discard a possible analysis bias due to metformin treatment of DM2 patients, a drug with potential genotoxicity. Although we do not intend to compare DM1 versus DM2 patients, a similar increase of nuclear abnormalities was observed in both groups, suggesting than differences between controls and DM1 are indeed significant despite the small sample size of DM1 group (Table 2).

In a similar work, Martinez-Perez et al. reported a higher number of micronuclei in blood of 15 DM2 Mexican patients taking metformin, in this case with sulfonylurea [48]. A cytogenetic report by Kulkarni et al. also showed an increased structural chromosomal aberrations of peripheral blood leucocyte in DM2 patients from Bombay, India, treated with Chlorpropamide [49]. None of these studies included a direct comparison with DM1 patients as we did.

Our results showed increased frequencies of nuclear abnormalities in DM patients; therefore, BMCyt could serve as a fast and easily accessible test to assess genotoxic damage during dental visits of DM patients, helping to monitor their disease.

## 4. Conclusions

In our study, quantitative BMCyt assay of epithelial cells from cheek and tongue showed significantly higher number of micronuclei and other abnormalities in both DM1 and DM2 patients, as compared to healthy control group. Implementation of BMCyt assay, at clinical level during dental care visits, would help enhance the quality of management for patients with diabetes and its complications.

## Figures and Tables

**Table 1:** Characteristics of controls subjects and patients with DM1 and DM2.

Variable	Controls	SD	DM1	SD	*P*	DM2	SD	*P*
	(*n* = 40)		(*n* = 10)			(*n* = 40)		
Female (%)	72.5	-	50.0		0.360	75.0	-	0.360
Age (yr)	51.2	12.3	42.0	9.7	0.131	57.0	13.8	0.141
Body Mass Index (kg/m^2^)	26.2	3.9	24.5	3.2	1.000	32.8	12.0	0.002
Disease duration (yr)	-	-	9.5	6.9	-	12.2	9.0	-
Soda intake (%)	87.5	-	20.0	-	0.001	47.5	-	0.001
Brushing (%)	82.5	-	40.0	-	0.006	72.5	-	0.284
Dental floss (%)	27.5	-	20.0	-	0.629	0.0	-	0.001
Mouth wash (%)	32.5	-	20.0	-	0.440	5.0	-	0.002
Employed (%)	30.0	-	50.0	-	0.232	22.5	-	0.446
High school (%)	25.0	-	30.0	-	0.747	10.0	-	0.077

Values given as percentage (%) or mean ± standard deviation (sd); *P*, *P* value.

**Table 2:** Nuclear cells characteristic (mean%  ± SD) in controls and patients with DM1 and DM2.

Group	MN			NB			KL			KR			PN			CC			DIF		
	*f*	SD	*P*	*f*	SD	*P*	*f*	SD	*P*	*f*	SD	*P*	*f*	SD	*P*	*f*	SD	*P*	*f*	SD	*P*
*Buccal*																					
Controls	0.07	0.06		0.76	0.42		6.14	2.69		4.17	2.53		1.19	0.65		3.47	1.42		84.20	6.10	
DM1	0.75	0.31	0.001	1.24	0.48	0.846	23.99	5.54	0.001	14.38	6.13	0.001	1.80	0.70	0.762	15.98	3.11	0.001	41.88	12.02	0.001
DM2	0.52	0.27	0.001	2.40	1.80	0.001	19.40	7.72	0.001	10.02	5.39	0.001	4.04	2.10	0.001	6.00	3.58	0.001	57.62	16.16	0.001
*Lingual*																					
Controls	0.06	0.05		0.69	0.34		6.21	2.41		3.89	1.34		1.12	0.52		3.75	1.84		84.27	4.48	
DM1	0.81	0.22	0.001	1.33	0.42	0.088	25.81	3.56	0.001	14.78	5.22	0.001	1.28	0.55	1.000	13.41	4.14	0.001	42.59	6.32	0.001
DM2	0.41	0.21	0.001	1.67	1.17	0.001	18.66	8.12	0.001	8.77	4.11	0.001	3.22	1.62	0.001	6.27	3.90	0.002	61.01	14.22	0.001

*f*, frequency. SD, standard deviation. *P*, *P* value. MN, micronuclei. NB, nuclear buds. KL, karyolytic cells. KR, karyorrhexis. PN, pyknotic nucleus. CC, condensed chromatin. DIF, normal differentiated cell.
